# Synthesis of Copper Nanoparticles from Cu^2+^-Spiked Wastewater via Adsorptive Separation and Subsequent Chemical Reduction

**DOI:** 10.3390/nano11082051

**Published:** 2021-08-12

**Authors:** Hye-Jin Hong, Jungho Ryu

**Affiliations:** 1 Department of Environmental Engineering, Chungbuk National University, Chungdae-ro 1, Seowon-Gu, Cheongju 28644, Korea; hyejiny@chungbuk.ac.kr; 2 Geologic Environment Research Division, Korea Institute of Geoscience and Mineral Resources (KIGAM), Daejeon 34132, Korea

**Keywords:** cupric ion, copper nanoparticles, wastewater, adsorption, reduction

## Abstract

Copper in ionic form (Cu^2+^) should be removed from wastewater because of its harmful effects on human health. Meanwhile, Cu-metal nanoparticles (Cu^0^ NPs) are widely used in various applications such as catalysts, optical materials, sensors, and antibacterial agents. Here, we demonstrated the recovery of Cu^2+^ from wastewater and its subsequent transformation into Cu^0^ NPs, a value-added product, via continuous adsorption followed by chemical reduction by hydrazine. To separate and enrich Cu^2+^ from wastewater, a biosorbent that exhibits excellent selectivity and adsorption capacity toward Cu^2+^, i.e., polyethyleneimine-grafted cellulose nanofibril aerogel (PEI@CNF), was packed into a column and used to treat 20 mg/L Cu^2+^ wastewater at a flow rate of 5 mL/min. The Cu^2+^ adsorption reached equilibrium at 72 h, and the Cu^2+^-saturated column was eluted using 0.1 M of HCl. After five consecutive elutions of Cu^2+^ from the adsorbent column, a Cu^2+^-enriched solution with a concentration of 3212 mg/L was obtained. The recovered Cu^2+^ concentrate was chemically reduced to obtain Cu^0^ NPs by reaction with hydrazine as a reductant in the presence of sodium dodecyl sulfate (SDS) as a stabilizer. The solution pH and hydrazine/Cu^2+^ ratio strongly affected the reduction efficiency of Cu^2+^ ions. When 0.1 M of SDS was used, spherical 50–100 nm Cu^0^ NPs were obtained. The results demonstrate that Cu^2+^-spiked wastewater can be converted into Cu^0^ NPs as a value-added product via adsorption followed by chemical reduction.

## 1. Introduction

Copper (Cu^2+^) is a highly toxic heavy metal that should be removed from water. Because of its harmful effects on human health, the United States Environmental Protection Agency (USEPA) has regulated its concentration in drinking water to less than 1.3 mg/L [1]. Large amounts of wastewater produced in various industries contain Cu^2+^ at a concentration greater than 1.3 mg/L. Consequently, an appropriate treatment to remove Cu^2+^ from industrial wastewater should be developed.

Even though Cu^2+^ is considered a toxic pollutant in the aquatic environment in its ionic form, copper metal (Cu^0^) is a valuable resource widely used in various industrial applications. Because of its high electric conductivity and facile processibility, Cu^0^ is considered an essential element in various applications such as building construction, electronics, wires, motors, radiators, connectors, and automobiles. In the United States, 1.2 million tons of Cu^0^ were produced in 2020 [2]. 

The application of Cu^0^ has recently been extended to more value-added products such as catalysts, antibacterial agents, optical materials, and gas sensors [3,4,5,6]. To satisfy the performance standards for these products, the Cu^0^ particles should be nanoscale. Numerous studies have been conducted to synthesize Cu^0^ nanoparticles (Cu^0^ NPs). Chemical treatment, electrochemical, photochemical, sonochemical, and thermal treatment methods have been developed to prepare stable Cu^0^ NPs [7]. Among them, chemical reduction has been intensively investigated because of its facile procedure [8,9]. In this method, a reducing agent provides electrons for the reduction of Cu salts such as CuSO_4_, CuCl_2_, and Cu(NO_3_)_2_. Various reducing agents and capping agents have been introduced to prepare Cu^0^ NPs [8,10,11,12]. However, for the practical application of Cu^0^ NPs, the economics of the synthesis procedure and the compatibility with mass production methods must be considered. 

In the present study, we used wastewater containing Cu^2+^ as a raw material for the low-cost synthesis of Cu^0^ NPs. In general, the low Cu^2+^ concentration in wastewater (~100 mg/L) and the presence of other impurities in the waste solution make synthesizing Cu^0^ NPs from a waste stream by a direct chemical reduction method difficult [13,14].

To separate and simultaneously enrich Cu^2+^ from wastewater, we used a Cu^2+^-selective biosorbent, polyethyleneimine-grafted cellulose nanofibril aerogel (PEI@CNF aerogel), to treat wastewater. A 42.4 cm^3^ cylindrical PEI@CNF aerogel was first prepared and packed in a column module for continuous Cu^2+^ separation from wastewater. The Cu^2+^ adsorption and consecutive elution for Cu^2+^ enrichment were performed in continuous operation mode. Cu^0^ was obtained by the chemical reduction of Cu^2+^ enriched in the eluent using hydrazine as a reductant. To obtain Cu^0^ NPs, sodium dodecyl sulfate (SDS) was added to the eluent. The synthesis conditions for Cu^0^ NPs were systematically investigated in terms of solution pH, hydrazine/Cu^2+^ ratio, SDS concentration, and physicochemical properties of the Cu precipitates.

## 2. Materials and Methods

### 2.1. Materials

Copper(II) sulfate (CuSO_4_, reagent grade, Wako Chemicals, Tokyo, Japan) was used to prepare simulated Cu^2+^-containing wastewater. For the synthesis of the PEI@CNF aerogel, polyethyleneimine (PEI, Mw 60,000, Sigma-Aldrich, Merck, St. Louis, MO, USA) was used, along with cellulose nanofibrils (CNFs) from hard wood pulp, which were purchased from the University of Maine Process Development Center. Glutaraldehyde (25% in water, Sigma-Aldrich, Merck, St. Louis, MO, USA) was used for cross-linking the PEI@CNF aerogels. Hydrochloric acid (HCl, 36%, Junsei chemical Co., Ltd., Tokyo, Japan) was used to elute Cu^2+^ from the PEI@CNF aerogel. Sodium hydroxide (NaOH, 98%, Sigma-Aldrich, Merck, St. Louis, MO, USA) was used to adjust the solution pH. Hydrazine hydrate (N_2_H_4_, 50–60% in H_2_O, Sigma-Aldrich, Merck, St. Louis, MO, USA) was used as a reductant. To prepare nanosized Cu^0^ particles, SDS (≥95%, Sigma-Aldrich, Merck, St. Louis, MO, USA) was used as a stabilizer.

### 2.2. Preparation of PEI@CNF Aerogel

For the continuous separation and enrichment of Cu^2+^ from wastewater, PEI@CNF aerogel was prepared. The preparation procedure of the PEI@CNF aerogel has been well described elsewhere [15]. First, a CNF slurry (50 g) was thoroughly mixed with 2 g of PEI to obtain a homogeneous mixture. Twenty-five grams of the resultant mixture was placed in a plastic framework with a diameter of 1.4 cm and length of 11.5 cm, frozen at −20 °C in a deep freezer (FCG-150, Lab Companion) for 12 h, and then freeze-dried (Bonduiro, ilShin Biobase, Dongducheon, Republic of Korea) at −40 °C and 0.008 torr for 24 h. The freeze-drying process removed ice from the PEI@CNF mixture, leaving macropores. This process resulted in a sponge-like PEI@CNF mixed aerogel. After the plastic framework was removed, the PEI@CNF mixed aerogel was placed in a 2 wt% glutaraldehyde solution for chemical cross-linking. The cross-linking was completed after 1 h, and the PEI@CNF aerogel was subsequently washed with deionized (DI) water several times to remove unreacted glutaraldehyde on the aerogel.

### 2.3. Continuous Cu^2+^ Adsorption–Elution Experiments

**Figure 1:**
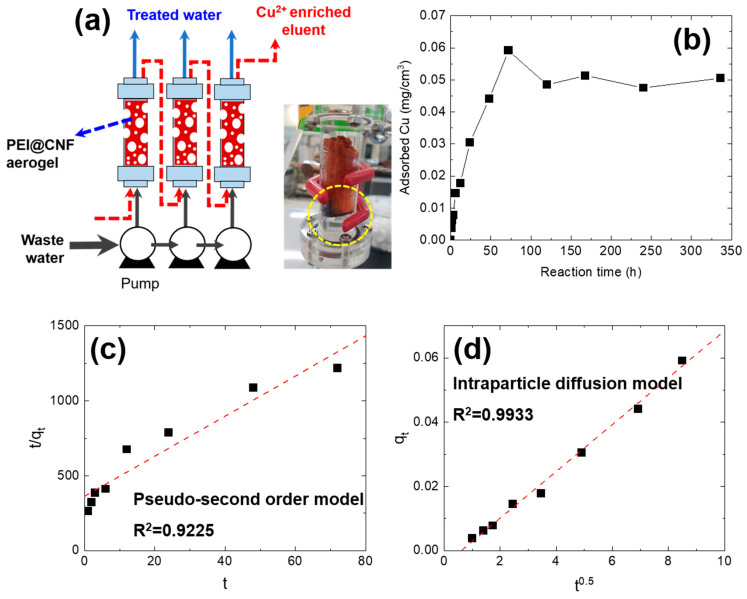
Continuous Cu^2+^ adsorption process in wastewater (**a**), the adsorbed amount of Cu^2+^ ions on the PEI@CNF aerogel column as a function of reaction time (**b**), the fitting result using a pseudo-second-order kinetic model (**c**), and the fitting result using the intraparticle diffusion model for the continuous Cu^2+^ adsorption process on the PEI@CNF aerogel column (**d**).

The continuous adsorption of Cu^2+^ was conducted using a column packed with the PEI@CNF aerogel. One piece of cylindrically shaped PEI@CNF aerogel (radius = 1.5 cm, height = 6 cm, 42.4 cm^3^) was packed in the acryl column, and simulated Cu^2+^ wastewater was supplied to the column in upflow mode at a flow rate of 5 mL/min using a peristaltic pump (EMP600, EMS tech, Yongin, Republic of Korea ). The configuration of the adsorption–elution module is described in Figure 1a. For simulated Cu^2+^-containing wastewater, a 20 mg/L Cu^2+^ solution was prepared using CuSO_4_ as a solute. To determine the saturation point of the PEI@CNF aerogel, the adsorption time was varied from 0 to 14 days.

The Cu^2+^ adsorbed onto the PEI@CNF aerogel was desorbed using 0.1 M of HCl (50 mL) solution as an eluent. The flow rate for elution was 5 mL/min in upflow mode. We used an eluent volume of 50 mL, which is similar to the volume of PEI@CNF aerogel used to enrich the Cu^2+^ in the eluent. For the further enrichment of Cu^2+^ in the eluent, consecutive Cu^2+^ desorptions were carried out with five different Cu^2+^-adsorbed PEI@CNF columns. The pH of the eluent was adjusted to 1 after each desorption cycle to ensure complete release of Cu^2+^ into the eluent. After five desorption cycles, a Cu^2+^-enriched solution was obtained. The concentration of Cu^2+^ in the wastewater was measured by atomic absorption spectroscopy (AAS, AA-7000, Perkin-Elmer, Waltham, USA). The adsorbed amount of Cu^2+^ per unit volume of the PEI@CNF aerogel in the column was evaluated by measuring the concentration of Cu^2+^ in the eluent. Equation (1) was used to calculate the adsorbed mass of Cu^2+^ (*q*):

(1) $$q\;\left(\frac{\mathrm{mg}}{{\mathrm{cm}}^{3}}\right)=\frac{C_{e}\times V_{\mathrm{eluent}}}{V_{\mathrm{aerogel}}}\;$$

where *C*_e_ (mg/L) is the concentration of Cu^2+^ in the eluent and *V*_eluent_ (L) and *V*_aerogel_ (cm^3^) are the volumes of the eluent and the PEI@CNF aerogel packed in the column, respectively.

### 2.4. Reduction of Cu^2+^ to Cu^0^ NPs

A 4000 mg/L Cu^2+^ solution was prepared using CuSO_4_ as a solute and 0.1 M of HCl as a solvent to mimic the final composition of the Cu^2+^-enriched eluent. Fifty milliliters of the Cu^2+^-enriched solution was used in reduction experiments. Chemical reduction of Cu^2+^ to Cu^0^ was conducted with hydrazine/Cu^2+^ ratios of 0.5, 1, and 2. The solution pH was varied from 1 to 6 to determine the optimal reduction condition. To control the size of the Cu^0^ NPs, 0–0.1 M of SDS was also added to the eluent. After chemical reduction for 5 min, a Cu precipitate was formed. The Cu precipitate was collected via centrifugation at 8000 rpm (Hanil, Supra 30K, Daejeon, Republic of Korea), washed twice with deionized water, and dried in an oven at 60 °C. All of the Cu reduction experiments were carried out in duplicate.

### 2.5. Characterization

The mechanical properties of the PEI@CNF aerogel were analyzed. The specific surface area was quantified via the Brunauer–Emmett–Teller (BET) technique with N_2_ adsorption–desorption isotherms obtained using a surface characterization analyzer (3Flex 3500, Micromeritics, Norcross, GA, USA). The PEI content was determined by measuring the amount of nitrogen (N) immobilized on the aerogel. CHNS elemental analysis was carried out using an automated elemental analyzer (Thermo, EA1112, FLASH2000). The compressive strength was measured using a universal testing machine (UTM, 5567A, Instron, Norwood, MA, USA) equipped with a 10 kN load cell. The PEI@CNF aerogel with an aspect (length-to-diameter) ratio smaller than 1.5 was compressed at room temperature until 70% strain using a constant crosshead speed of 5 mm/min. X-ray diffraction (XRD) analysis (SmartLab, Rigaku) was carried out to analyze the crystalline structure of the prepared Cu^0^ NPs. The diffraction data were collected over the 2*θ* range 10° ≤ 2*θ* ≤ 80°. The morphology of the Cu^0^ NPs was observed by scanning electron microscopy (SEM, NanoLab 650, FEI company, Hillsboro, OR, USA).

## 3. Results and Discussion

### 3.1. Enrichment of Cu^2+^ from Wastewater Using PEI@CNF Aerogel

The remediation of wastewater and Cu^2+^ recovery were simultaneously conducted using the PEI@CNF aerogel. Table 1 shows the basic properties of the PEI@CNF aerogel. The PEI@CNF aerogel has previously been reported to exhibit 120 mg/g (7.01 mg/cm^3^) of Cu^2+^ adsorption capacity and excellent selectivity toward Cu^2+^ in the presence of minerals such as Na^+^, K^+^, Mg^2+^, and Ca^2+^, as well as in the presence of metal ions such as Co^2+^, Ni^2+^, and Zn^2+^ [15]. Only Cu^2+^ was concentrated on the PEI@CNF aerogel because of chelate bond formation between Cu^2+^ ions and the amine groups of the PEI moiety on the CNF aerogel [15]. The PEI@CNF aerogel exhibited 33.9 wt% PEI grafting density with a highly porous morphology. In addition, the synthesized PEI@CNF aerogel was mechanically stable and versatile, with a compressive strength of 0.6 MPa and rapid recovery of its shape when the compression stress was removed. The excellent Cu^2+^ adsorption behavior, good mechanical stability of the aerogel, and facile synthesis procedure that is compatible with mass production methods support the feasibility of PEI@CNF aerogel for the treatment of Cu^2+^-contaminated wastewater. 

We prepared cylindrical-shaped PEI@CNF aerogel samples with a volume of 42.4 cm^3^ (*r* = 1.5 cm, *h* = 6 cm) and inserted them into a column for the continuous treatment of Cu^2+^-contaminated wastewater. During continuous column adsorption, the treated effluent was pumped into the column, which resulted in a concentration of Cu^2+^ on the PEI@CNF aerogel. The Cu^2+^ recovery was confirmed by a color change of the PEI@CNF aerogel from red to blue, consistent with the color of Cu^2+^ ions (Figure 1a). To determine the saturation time of Cu^2+^ on the PEI@CNF aerogel, the contact time with Cu^2+^ wastewater was varied from 0 to 14 days and the flow rate was fixed at 5 mL/min. The amount of Cu^2+^ adsorbed onto the PEI@CNF aerogel proportionally increased until 72 h and reached equilibrium at ~0.05 mg/cm^3^. 

To analyze the Cu^2+^ adsorption behavior on the PEI@CNF aerogel during column operation, pseudo-second-order and intraparticle diffusion kinetic models were used to fit the Cu^2+^ adsorption data collected over a period of 72 h (Figure 1c,d, also see Supporting Information). The Cu^2+^ adsorption rate during the first 72 h was well fitted using the intraparticle diffusion model (R2 = 0.9933), indicating that the rate of Cu^2+^ adsorption onto the PEI@CNF aerogel was controlled by the diffusion of Cu^2+^ ions in the pores of the PEI@CNF aerogel. This result differs from that of batch adsorption experiments in a previous study [15]. In batch adsorption experiments, Cu^2+^ adsorption data were well fitted using a pseudo-second-order model (Table S1). Because the flow rate was only 5 mL/min during continuous column operation in the present study, the flow rate was apparently insufficient to enable the effective diffusion of wastewater into the mesopores of the PEI@CNF aerogel. In addition, the relatively low initial Cu^2+^ concentration (~20 mg/L) compared with that used in the previously reported batch operation (~200 mg/L) led to a substantially lower diffusion rate of wastewater into the PEI@CNF aerogel. On the basis of this result, the optimal continuous Cu^2+^ adsorption reaction time was determined to be 72 h.

**Figure 2:**
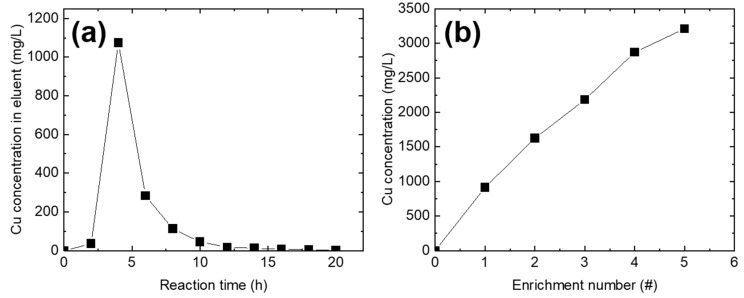
Elution profile of Cu^2+^ using 0.1 M of HCl (**a**) and the Cu^2+^ concentration in the eluent as a function of the number of enrichment cycles (**b**).

To demonstrate the enrichment of Cu^2+^ ions on the Cu^2+^-adsorbed PEI@CNF aerogel (for 72 h), Cu^2+^ ions were desorbed using a 0.1 M HCl solution (Figure 2a). When the solution pH was lowered to 1 (0.1 M of HCl), amine groups on the PEI@CNF aerogel were protonated, which led to the destruction of the chelate bond between the PEI and Cu^2+^ ions, resulting in the release of Cu^2+^ [15]. When 0.1 M of HCl was used as the eluent, Cu^2+^ was rapidly desorbed into the medium and the desorption was completed with a 1.4 bed volume (~60 mL) of 0.1 M of HCl. Unlike the Cu^2+^ adsorption reaction, the Cu^2+^ elution was rapidly completed within 15 min. For the complete desorption and enrichment of Cu^2+^ on the PEI@CNF aerogel, 50 mL of 0.1 M of HCl was used as an optimal volume of eluent and was circulated for 1 h. The obtained eluent exhibited a Cu^2+^ concentration of ~900 mg/L.

For further enrichment of the Cu^2+^ ions, five sequential Cu^2+^ elutions were conducted with five different Cu^2+^ adsorbed PEI@CNF aerogels. In the first cycle, an eluent Cu^2+^ concentration of 900 mg/L was obtained. In the second cycle, approximately 500 mg/L of Cu^2+^ desorption from the PEI@CNF aerogel was achieved despite the pH adjustment. Even though the Cu^2+^ desorption efficiency moderately decreased, Cu^2+^ could be enriched to a concentration of 3212.08 mg/L after five consecutive elution cycles (Figure 2b). Relative to the initial concentration of the simulated wastewater (~20 mg/L Cu^2+^), the Cu^2+^ was concentrated 160-fold after the PEI@CNF aerogel adsorption process. 

### 3.2. Reduction of Cu^2+^ to Cu^0^ NPs by Hydrazine

The Cu^2+^ ions in 0.1 M of HCl eluent were converted to Cu^0^ NPs using hydrazine as a reductant. The oxidation–reduction reaction is described as follows:

N_2_H_4_ + 4OH^−^ → N_2_ + 4H_2_O + 4e^−^ + 1.16 (*E*^0^/*V*) (2)

Cu^2+^(aq) + 2e^−^ → Cu(s) 0.34 (*E*^0^/*V*) (3)

Hydrazine is a powerful reductant with a standard reduction potential of +1.16 V. With regard to the reduction potential difference between hydrazine and Cu^2+^, hydrazine can reduce Cu^2+^ to Cu^0^. Except hydrazine and Cu^2+^, the concentrated Cl^−^ ions are present in the eluent (0.1 M of HCl). Owing to the high ionization property of Cu^2+^ and Cl^−^, these ions barely form CuCl_2_ precipitate under ambient conditions. Otherwise, hydrazine is a kind of basic ligand that forms coordination complexes with transition metal cations such as Cu^2+^ and reduces them to a metal [16]. It is reported that the hydrazine-Cu^2+^-Cl^−^ complexes such as (N_2_H_4_)CuCl, (N_2_H_5_)_2_Cu_3_Cl_6_, (N_2_H_5_)CuCl_4_∙2H_2_O, and (N_2_H_5_)CuCl_3_ can be formed in the presence of Cl^−^ [17]. However, these precipitates were not observed in this study. Various hydrazine/Cu^2+^ ratios were tested to optimize the conditions for synthesizing Cu^0^ NPs according to the electron balance of the oxidation–reduction reaction between Cu^2+^ and hydrazine (Equations (2) and (3)).

In addition, the solution pH was varied from 1 to 6. Because Cu^2+^ forms a precipitate (Cu(OH)_2_) at pH > 6, a pH greater than 7 is inappropriate for the synthesis of Cu^0^ NPs via a bottom-up synthesis approach. 

**Figure 3:**
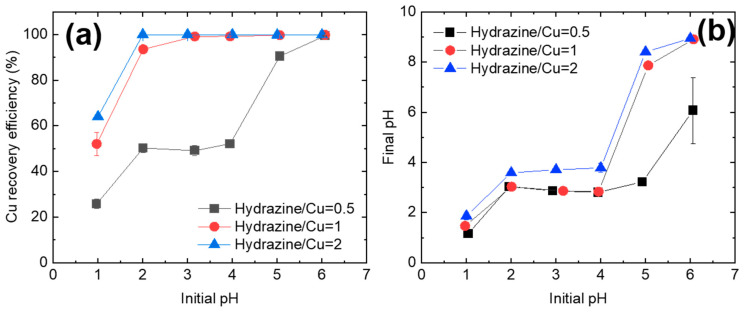
Recovery efficiency of Cu (**a**) and the pH change of solution (**b**) as functions of the initial pH, as tested for various hydrazine/Cu ratios.

After hydrazine addition, the Cu^2+^ was rapidly reduced and recovered as precipitates. Irrespective of the hydrazine/Cu^2+^ ratio, the Cu precipitation efficiency increased with the solution pH (Figure 3a). The Cu^2+^ readily formed precipitates at slightly acidic or neutral pH but not at pH 1. At a hydrazine/Cu ratio of 2, 100% Cu precipitation occurred (~100% efficiency) over a wide pH range (i.e., 2 ≤ pH ≤ 6). When the hydrazine/Cu^2+^ ratio was reduced to 1, the pH range over which a 100% Cu precipitation efficiency was achieved slightly decreased to 3 ≤ pH ≤ 6. Finally, a 100% Cu recovery was obtained at only pH 6 when the hydrazine/Cu^2+^ ratio was 0.5. Although an increase in the hydrazine/Cu^2+^ ratio improved the Cu precipitation efficiency, the factor that most strongly affected the Cu precipitation efficiency was the solution pH. At pH 1, Cu precipitation efficiencies of only 25.8%, 52.1%, and 64.0% were achieved at hydrazine/Cu^2+^ ratios of 0.5, 1, and 2, respectively. Meanwhile, a 100% Cu precipitation was achieved at pH 6 irrespective of the hydrazine/Cu^2+^ ratio.

Figure 3b shows the change in solution pH during the Cu^2+^ reduction reaction. When the initial pH was in the range of 1–4, the solution pH was similar after the reduction–precipitation reaction. By contrast, when the initial solution pH was 5–6, the pH after reaction was in the alkaline range (8 ≤ pH ≤ 10). Although a 100% Cu precipitation efficiency was attained at 3 ≤ pH ≤ 6 (hydrazine/Cu ratio = 1, 2), the Cu recovery mechanisms at 3 ≤ pH ≤ 4 and 5 ≤ pH ≤ 6 differ. 

**Figure 4:**
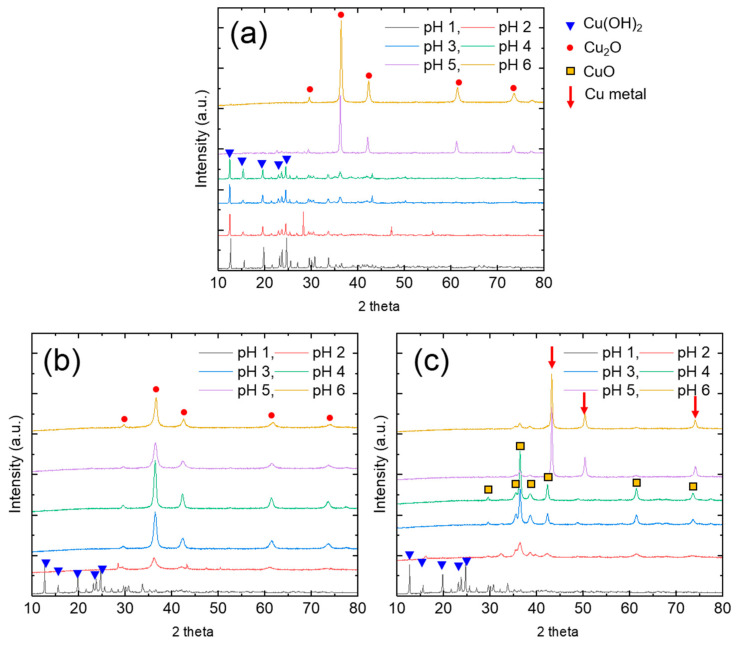
XRD patterns of recovered Cu precipitates obtained at hydrazine/Cu^2+^ ratios of 0.5 (**a**), 1 (**b**), and 2 (**c**).

To determine the chemical state of the recovered Cu precipitates, we carried out XRD analysis (Figure 4). The results show that, at pH 1, the Cu was recovered as copper(II) hydroxide (Cu(OH)_2_). This result means that hydrazine did not reduce the Cu^2+^ at pH 1. Hydrazine can be protonated depending on the solution pH, thereby changing its form to ionized hydrazinium ions (N_2_H_5_^+^ and N_2_H_6_^2+^) via the following reactions Equations (4) and (5) [18]:

N_2_H_4_ + H_2_O ⇌ N_2_H_5_^+^ + OH^−^ *K_b_*: 8.5 × 10^−7^ (4)

N_2_H_5_^+^ + H_2_O ⇌ N_2_H_6_^2+^ + OH^−^ *K_b_*: 8.9 × 10^−16^ (5)

where *K*_b_ is the corresponding base dissociation constant. Thus, N_2_H_6_^2+^ and N_2_H_5_^+^ ions can exist at pH 1, with a negligible concentration of N_2_H_4_. Because hydrazinium ions have a much weaker reduction potential (0.23 V) than hydrazine (1.16 V), Cu^2+^ reduction occurs more effectively under alkaline conditions [19]. At pH 1, hydrazine is protonated and converted to hydrazinium ions, simultaneously producing hydroxyl ions (OH^−^) (Equations (4) and (5)). Under this condition, Cu precipitation occurs via reaction with OH^−^ to form Cu(OH)_2_. The consumption of OH^−^ by this reaction leads to the solution pH being maintained at 1. At a hydrazine/Cu^2+^ ratio of 0.5, only Cu(OH)_2_ precipitate was obtained at 1 ≤ pH ≤ 4. At pH 5, sharp cuprite (Cu_2_O) peaks appeared in the XRD pattern, indicating that Cu^2+^ was reduced to Cu^+^. The lower oxidation state (+1) indicates that a relatively small amount of OH^−^ was consumed, resulting in a substantial increase in the solution pH. At a hydrazine/Cu^2+^ ratio of 1, the main peak of Cu_2_O appeared in the XRD patterns of the products obtained over a wide pH range (2 ≤ pH ≤ 6). However, the reduction reaction was insufficient to produce Cu^0^. At a hydrazine/Cu^2+^ ratio of 2, Cu(OH)_2_ was obtained at pH 1, and a CuO (oxidation state +2) and Cu_2_O (oxidation state +1) mixed phase was obtained at 2 ≤ pH ≤ 4. Finally, metallic Cu^0^ peaks appeared in the patterns of the products obtained at pH 5 and 6. Although the Cu precipitation efficiency was almost 100% at pH 6, the finally obtained Cu precipitates exhibited different phases depending on the hydrazine/Cu^2+^ ratio. Accordingly, the optimized condition for the recovery of Cu^0^ precipitates was determined to be a hydrazine/Cu^2+^ ratio of 2 and a pH of 6. 

**Figure 5:**
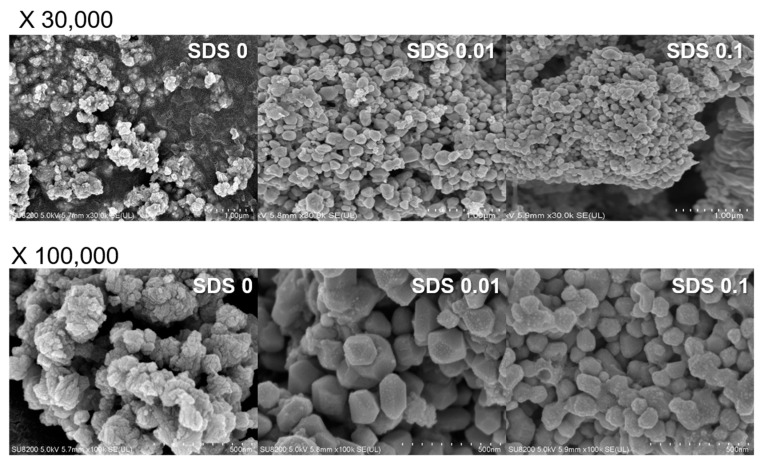
SEM images of Cu^0^ prepared in the absence and presence of SDS (0, 0.01, and 0.1 M).

Figure 5 shows SEM micrographs of the Cu^0^ precipitates recovered at a hydrazine/Cu^2+^ ratio of 2 and at pH 6 when SDS was added as a stabilizer. SDS is an anionic surfactant with a polar (hydrophilic) head group (SO_4_^2−^) and a hydrophobic hydrocarbon chain. The SDS self-assembles because of its amphiphilic nature, forming micelles at concentrations greater than the critical micelle concentration (CMC) and consequently providing a spherical template for Cu^2+^ ions [10]. In addition, SDS micelles prevent the oxidation of the surface of Cu^0^ NPs by blocking the transfer of oxygen. In the absence of SDS, Cu^0^ formed irregular agglomerates with a specific patterned morphology. When the SDS concentration was increased to 0.01 M, relatively spherical and 200–300 nm Cu^0^ particles were obtained. Given that the CMC of SDS is 0.008–0.010 M, the SDS micelles function as a template to form nanosized Cu^0^ particles [20]. When the SDS concentration was increased to 0.1 M, the size of the Cu^0^ NPs was reduced to 50–100 nm and the Cu^0^ NP agglomerates exhibited a more regular morphology. These results demonstrate that Cu^0^ NPs (~50–100 nm) can be prepared from Cu^2+^-spiked wastewater as a resource via adsorptive separation followed by chemical precipitation. 

## 4. Conclusions

Cu^0^ NPs are a useful material in various industries because of their excellent physicochemical properties. However, a more economical and mass-production-compatible method to prepare Cu^0^ NPs is needed to promote their practical application. In the present study, Cu^0^ NPs were prepared from Cu^2+^-spiked wastewater via continuous adsorption followed by chemical reduction. To separate and concentrate the Cu^2+^ ions from the wastewater, a biosorbent that demonstrates excellent selectivity and a high adsorption capacity toward Cu^2+^, i.e., PEI@CNF aerogel, was used. During the continuous adsorption process in a column, when 20 mg/L of Cu^2+^ wastewater was fed at a flow rate of 5 mL/min, adsorption equilibrium was achieved in 72 h. An intraparticle diffusion kinetic model showed good agreement with the Cu^2+^ adsorption rate in continuous operation mode. The Cu^2+^ adsorbed onto the sorbent was subsequently eluted using 0.1 M of HCl. After five consecutive elutions of Cu^2+^ from the sorbent, an enriched solution with a Cu^2+^ concentration of 3212 mg/L was obtained. Chemical reduction was carried out by adding hydrazine and SDS as a reductant and a stabilizer, respectively, to the obtained Cu^2+^-enriched solution. The solution pH and hydrazine/Cu^2+^ ratio strongly affected the reduction efficiency of Cu^2+^. Pure Cu^0^ NPs were obtained at pH 6 when the hydrazine/Cu^2+^ ratio was 2. When 0.1 M of SDS was added, spherical and 50–100 nm Cu^0^ NPs were obtained. These results demonstrate that Cu^2+^-spiked wastewater that requires remediation can be converted into Cu^0^ NPs as a value-added product via adsorptive separation and subsequent chemical reduction. 

## Figures and Tables

**Table 1:** Properties of the PEI@CNF aerogel [15].

Property		
PEI content (wt%)		33.9
Compressive strength (MPa)		0.6
BET surface area (m^2^/g)		3.69
Cu^2+^ adsorption capacity	(mg/g)	~120
	(mg/cm^3^)	7.01

## Supplementary Materials

The following are available online at https://www.mdpi.com/article/10.3390/nano11082051/s1, Table S1: Cu^2+^ adsorption kinetic model parameters on PEI@CNF aerogel in batch and column operation mode
