# Supplementary material for: Synthesis of Copper Nanoparticles from Cu2+-Spiked Wastewater via Adsorptive Separation and Subsequent Chemical Reduction
Source: Nanomaterials (Basel). 2021 Aug 12;11(8):2051. doi: 10.3390/nano11082051 (PMC8401681; doi:10.3390/nano11082051)
Supplement: Supplementary file 1 [file nanomaterials-11-02051-s001.zip › nanomaterials-1311923-supplementary.pdf]

Supplementary information

Synthesis of Copper Nanoparticles from Cu^{2+}-Spiked Wastewater via Adsorptive Separation and Subsequent Chemical Reduction

Hye-Jin Hong [1] and Jungho Ryu [2,*]

[1] Department of Environmental Engineering, Chungbuk National University, Chungdae-ro 1, Seowon-Gu, Cheongju, Chungbuk 28644, Korea; hyejiny@chungbuk.ac.kr
[2] Geologic Environment Research Division, Korea Institute of Geoscience and Mineral Resources (KIGAM), Daejeon 34132, Korea
* Correspondence: jryu@kigam.re.kr, +82-42-868-3943

[Adsorption Kinetic Models]

Pseudo-second order adsorption kinetic model is described as follows:

$$\frac{t}{q_t} = \frac{1}{k_2 q_e^2} + \frac{1}{q_e} t, \qquad (1)$$

where Q_t and Q_e (mg/cm^3) is adsorbed Cu^{2+} on PEI-CNF aerogels at time *t* (h) and equilibrium, respectively. k_2 represents pseudo-second order adsorption kinetic constants. *h* is the value reflects the initial adsorption rate on the adsorbent.

Intra-particle diffusion model equation can be expressed:

$$q_t = k_p t^{0.5} + X_i \qquad (2)$$

where k_p represents reaction rate constant and X_i is related to thickness of the boundary layer.

Table S1. Cu^{2+} adsorption kinetic model parameters on PEI@CNF aerogel in batch and column operation mode

	**Pseudo-second order model**			**Intraparticle diffusion model**		
	q_e	k_2	R^2	X_i	k_p	R^2
**Batch adsorption test**	3.94	0.204	0.9996	1.519	0.431	0.7334
**Continuous adsorption test**	0.0748	0.4919	0.9225	0.004	0.0073	0.9933
